# Music Training Program: A Method Based on Language Development and Principles of Neuroscience to Optimize Speech and Language Skills in Hearing-Impaired Children

**Published:** 2013

**Authors:** Samaneh Sadat Dastgheib, Mina Riyassi, Maryam Anvari, Hamid Tayarani Niknejad, Masumeh Hoseini, Mohsen Rajati, Mohammad Mahdi Ghasemi

**Affiliations:** 1 *Shefa Neuroscience Research Center, Tehran, Iran.*; 2 *Sinus and Surgical Endoscopic Research Center, Mashhad University of Medical Sciences, Mashhad, Iran.*; 3 *Speech and Language Rehabilitation Center (Shenavagostar)*

**Keywords:** Cochlear implantation, Hearing loss, Language development, Music therapy, Neuroscience

## Abstract

**Introduction:**

In recent years, music has been employed in many intervention and rehabilitation program to enhance cognitive abilities in patients. Numerous researches show that music therapy can help improving language skills in patients including hearing impaired. In this study, a new method of music training is introduced based on principles of neuroscience and capabilities of Persian language to optimize language development in deaf children after implantation.

**Materials and Methods:**

The candidate children are classified in three groups according to their hearing age and language development. The music training program is established and centered on four principles, as follows: hearing and listening to music (with special attention to boost hearing), singing, rhythmic movements with music and playing musical instruments.

**Results:**

Recently much research has demonstrated that even after cochlear implant operation, a child cannot acquire language to the same level of detail as a normal child. As a result of this study music could compensate this developmental delay .It is known that the greater the area of the brain that is activated, the more synaptic learning and plasticity changes occur in that specific area. According to the principles of neural plasticity, music could improve language skills by activating the same areas for language processing in the brain.

**Conclusion:**

In conclusion, the effects of music on the human brain seem to be very promising and therapeutic in various types of disorders and conditions, including cochlear implantation.

## Introduction

Music is an integral part of every culture, constantly weaving in and out of daily life. It is documented that the human brain is “hard-wired” for music; thus providing a biological basis for the importance of music in the human experience (1). The impact of music in children’s lives may be demonstrated via children’s literature in different languages, through lullabies and traditional plays. Recent studies have reported many benefits of music therapy in managing different diseases, particularly in disorders related to the central nervous system (2–5). 

Widespread research has studied the impact of music on the brain and its role in neural cell growth. The process of music interpretation in the human brain is very similar to that of language processing. Indeed, many language areas overlap with those of music. The basic concepts of music such as frequency, intensity, and tension levels are perceived via the primary auditory area, while the higher concepts such as musical phrases are processed in the secondary and association areas which closely overlap areas of language (1-4). Thus triggering the initial hearing and language processing centers with the use of music may engage cochlear-implant children with language concepts so that they can learn earlier and in a more natural way. In this paper we describe an educational method based on language development processing and advances in cognitive neuroscience to help children with impaired hearing in language acquisition. 

## Materials and Methods

The music training program established by the authors is centered on four principles, as follows:

1.Hearing and listening to music (with special attention to boost hearing)

2. Singing

3. Rhythmic movements with music

4. Playing musical instruments

It is recommended that children who are candidates for this method of rehabilitation are classified into three subgroups according to their hearing age and level of language development. These subgroups are:

**A.** These children have a hearing age of 0–1 year. According to their language development, these children are able to hear and listen to environmental and speech sounds. They also have the ability to separate speech sounds from non-speech sounds, but their ability to discriminate among all the sounds of their mother-tongue language is not as evident as in their normal peers. Children in this group are sensitive to a number of prosodic features of their language, such as vowel duration and pitch peaks. Based on their hearing age, these children may perceive some patterns and segments of sounds in syllabic structures and understand some limited words. Details of the individualized music training program for these children are provided (Table 1). 

**B.** These children have a hearing age of 2, 3, or 4 years. They have considerable experience of sounds, and are able to detect, discriminate, identify, and recognize them. Their ability to identify and recognize meaningful words in closed and open-set is fully evident. These children are also able to understand single words in complicated language patterns such as phrases and simple sentences, but they fail to understand the entire phrase or simple sentence. Details of the individualized music training program for these children are provided (Table 2). 

**C.** These children have a hearing age of 5, 6, or 7 years. Children in this group cannot only understand and follow meaningful words in many phrases, simple, and complicated sentences but they can also perceive most parts of these sentences. However, the children in this group cannot coordinate between more complex language structures and the situations in which every structure is used (lack of pragmatic skills). Details of the individualized music training program for these children are provided (Table 3).

**Table1:** Musical Training Program Individu- alized for Subgroup A

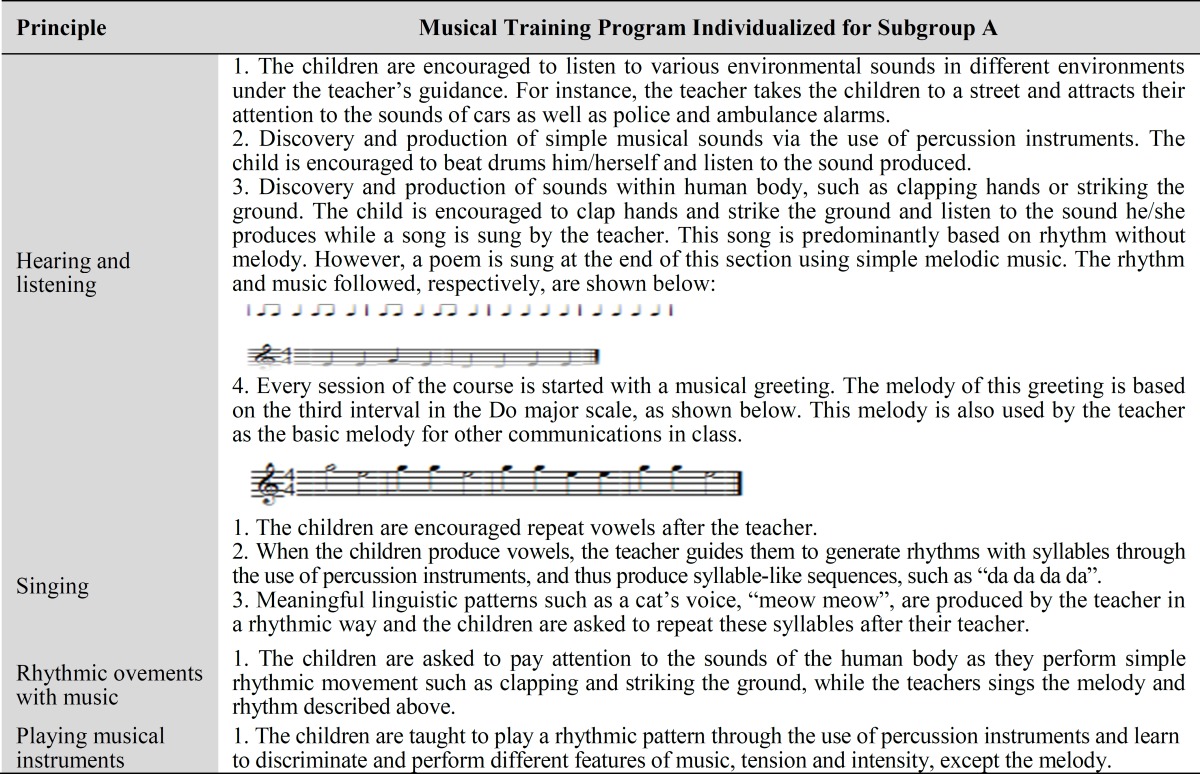

**Table 2:** Musical Training Program Individualized for Subgroup B

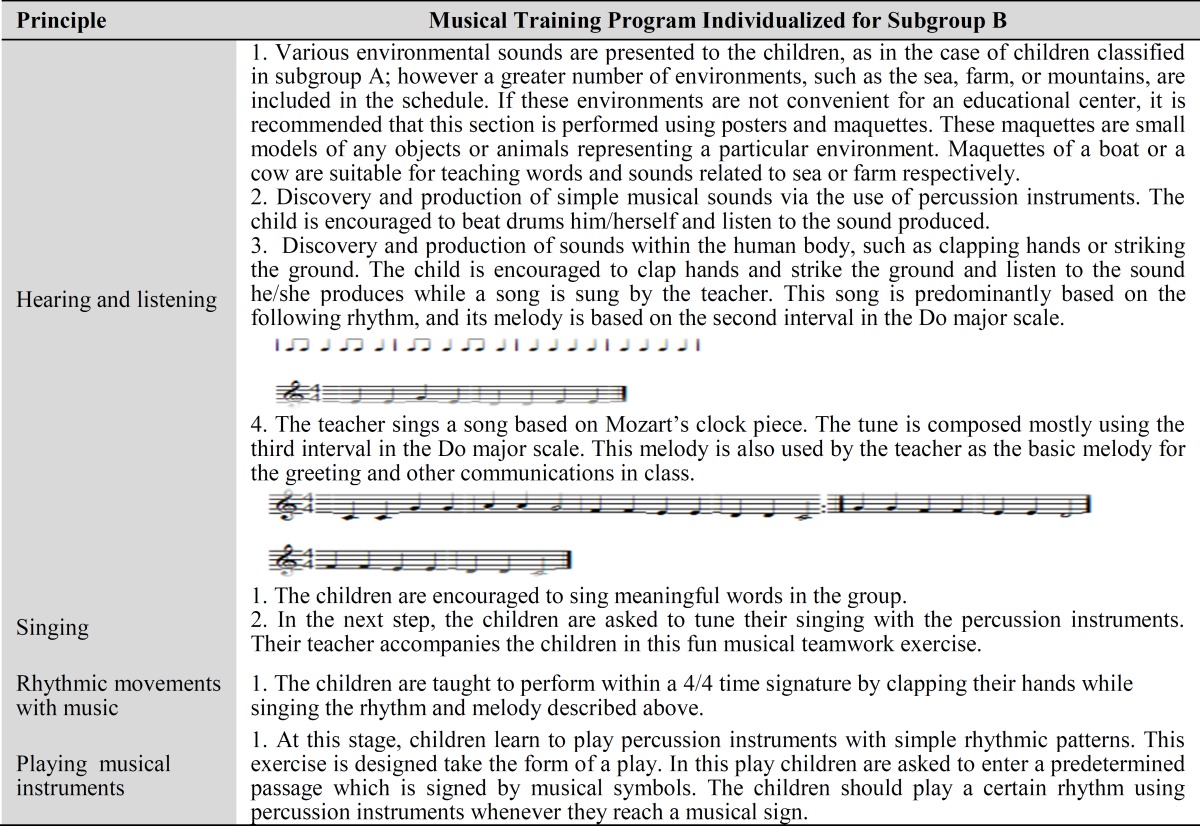

**Table 3:** Musical Training Program Individualized for Subroup C

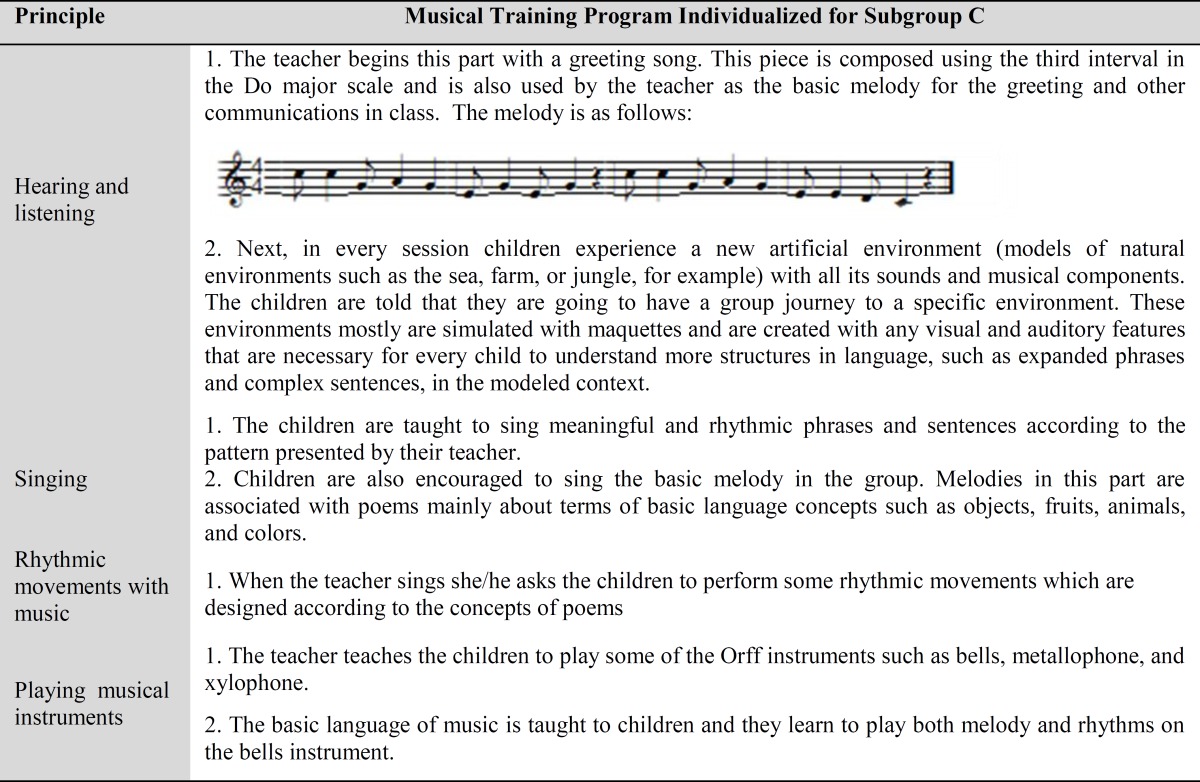

## Discussion

The cochlear implant, one of the most successful biological prosthesis over the last 40 years, has improved the auditory perception of complete or partially deaf children by up to 95 percent. Improvement of audition in these children allows the development of both complex and simple linguistic skills. In normal children these skills are developed during the first years of life, but in deaf children there is a developmental delay in language ability due to impaired audition. Audition has the greatest impact on language acquisition (6). The child at first hears a combination of environmental sounds and human speech. As time passes, he/she gradually develops the ability to make a phonic relationship between what he/she heard and what he/she learned. It is important to note that the language developmental process in normal children is far removed from that in partially or completely deaf children, since impaired hearing in these children prevents them from developing a rich auditory store. Literature shows that a child can discriminate among different linguistic patterns 1 year after a cochlear implant operation, but the most important consideration is how the child will communicate and how the language will benefit in the long term, rather than the communication itself. Much research and personal experience among rehabilitation specialists has demonstrated that even after cochlear implant operation, a child cannot acquire language to the same level of detail as a normal child. Many factors are involved in how a child with impaired hearing acquires language after a cochlear implant operation, including the age at which the operation is performed, the number of active electrodes, the duration of deafness, and the type of rehabilitation (7). 

As already mentioned, the current method is provided based on the language developmental process. It is known that children with hearing loss have a developmental delay in their language acquisition, but no other disorder of language is observed (8). The major issue is that for many candidates of cochlear implant operation, the perfect age in terms of brain plasticity for native language acquisition of 0–3 years has already passed (9,10). In the current method, the authors recommend that cochlear-implant children be approached with the goal of compensating for this delay through the use of music in their education.

At birth, and even before birth, humans are prepared to acquire and appropriate the language and speech of their environment. Early interactions with this environment give individuals the opportunity to process sounds and to maximize language acquisition and production. Infants demonstrate a range of speech perception abilities before they can produce any structures of their first language. Speech perception research has revealed that these abilities not only provide the basis for learning native-language sound categories, but also the basis for learning syllable structure and segmenting and storing of words. Phylogeny, embryology, and the interaction between human biological mechanisms and their environment are connected to auditory perception from the time sounds reach the organism in uterus through to 12 months of age, when the first words are generally uttered. Language acquisition studies show that newborns prefer their mother’s voice to the voices of other females. They also prefer to listen to infant directed speech (baby talk) which has a higher pitch, longer vowels, wider pitch variation and increased rhythmicity compared with adult-directed speech (11).

The neural mechanism underlying the language process is very similar to the process of language development. Auditory information is transformed from the medial geniculate body of the thalamus to the Heschl gyrus after a very primary process in the brain stem nuclei such as the inferior colliculus, inferior olive, and cochlear nuclei. The Heschl gyrus, which is known as the primary auditory cortex and is located in the depth of the lateral sulcus, is tonotopically organized. When the auditory data reach this area, the first awareness of hearing is perceived. In the next steps the simply processed data from the Heschl gyrus are passed to secondary and association auditory areas in a hierarchical pattern. Thus, the simple patterns of language, free morphemes, and bound morphemes are interpreted prior to more complex features such as words and sentences (12). Interestingly, the brain areas known for the interpretation of music closely overlap with the areas specified for language processing. Examples include the Broca area which is involved in processing language grammar and in planning for motor aspects of language such as organizing the laryngeal muscles to produce vowels and constants (13). Recent studies using the Event Related Potential (ERP) technique have shown that the Broca area functions as the main area for processing the syntax of music as well as language (13). It is known that the greater the area of the brain that is activated, the more synaptic learning and plasticity changes occur in that specific area (12). According to the principles of neural plasticity, music could improve language skills by activating the same areas for language processing in the brain. The results of various studies confirm this hypothesis. The impact of music therapy has been significant in patients with different language disorders, including aphasia and dyslexia (14,15). Studies on patients who use cochlear implants demonstrate that, like normal children, children with cochlear implants prefer singing to silence and may use musical stimulations as cues for linguistic recognition (9,16–18). 

Musical training has many advantages compared with passive music therapy because children are particularly involved during music training courses, engaging their brains with various activities ranging from singing to playing instruments.As expressed in one of the main principles in neuroplasticity, the “Donald Hebb rule”, neurons that fire together, wire together (19). Thus, motor, linguistic, and cognitive processes are better coordinated in children who participate in a music training course. Furthermore, since these children learn music within a group, their emotional and cognitive abilities are also improved. Experiencing problems in communicating with peers is one of the most painful issues that cochlear-implant children have to face. This prevents them from making friends and leaves them isolated among normal children. The fewer friends a child has, the less he speaks and plays. This is a vicious cycle, since the less a child speaks the less he can improve his communicative and linguistic skills. 

In research conducted to date, many clinical studies have demonstrated the benefit of music in the improvement of cognitive, behavioral, language, and even motor disorders such as dementia, autism spectrum disorders, attention deficit hyperactivity disorder, Parkinson’s disease, and aphasia (3,14,20–22). 

Among the different types of music studied, Mozart’s works have been considered to be more effective in comparison with works of all other composers, such that a phenomenon known as “Mozart’s effect” which refers to an enhancement of performance or a change in neurophysiological activity associated with listening to Mozart music was established (23–25).

Animal studies have also confirmed the findings of these clinical studies in regard to the therapeutic effects of music. Prenatal exposure to Mozart’s music increases neurogenesis and dendritic branching in the hippocampus and enhances the spatial learning ability in the offspring of rats (26). 

In the method presented in this study, almost all of the melodies are based on simple musical works by Mozart. 

## Conclusion

 In conclusion, the effects of music on the human brain seem to be very promising and therapeutic in various types of disorders and conditions, including cochlear implantation. The method described in this study is based on recent research on language development and neuroscience of music. However, very few studies have been performed in this field. There is currently no randomized controlled trial published which predominantly investigates the impact of music training in children using cochlear implants. Thus, randomized controlled trials with an adequate sampling method are highly recommended.
